# Bronchoscopic visualization of the inner cavity caused by *Pasteurella multocida* infection

**DOI:** 10.1016/j.heliyon.2023.e18588

**Published:** 2023-07-22

**Authors:** Kazutoshi Toriyama, Reimi Mizushima, Takashi Okuma, Yukihisa Takeda, Yusuke Watanabe, Hiroyuki Nakamura, Kazutetsu Aoshiba

**Affiliations:** a Department of Respiratory Medicine, Tokyo Medical University Ibaraki Medical Center, 3-20-1 Chuou, Ami, Inashiki, Ibaraki, 300-0395, Japan; b Department of Respiratory Medicine, Tokyo Medical University Hospital, 6-7-1 Nishishinjuku, Shinjuku-ku, Tokyo, 160-0023, Japan; c Department of Infectious Disease, Tokyo Medical University Hospital, 6-7-1 Nishishinjuku, Shinjuku-ku, Tokyo, 160-0023, Japan

**Keywords:** *Pasteurella multocida*, Pneumonia, Lung abscess, Cavity, Bronchoscopy

## Abstract

We report a 69-year-old man who presented to our hospital with cough and sputum production. He had been in close contact with six domestic cats. He had a smoking history of 40 pack-years and had been in close contact with six domestic cats. A chest computed tomography scan revealed multiple consolidations with cavities in both lung fields. *Pasteurella multocida* was cultured from his sputum*.* On bronchoscopic evaluation, the flexible bronchoscope was navigated through the right middle lobe bronchus, which opened inside the cavity, allowing visualization of a spider-web-appearing architecture consisting of many cord-like lung tissues loosely adherent to the cavity lumen. Using these findings, a diagnosis of cavity formation was made secondary to *Pasteurella multocida* infection. *Pasteurella* infection should be considered as a cause of a lung cavity in patients with chronic lung disease. History taking regarding animal exposure is important for its diagnosis.

## 1 Introduction

**Fig. 1:**
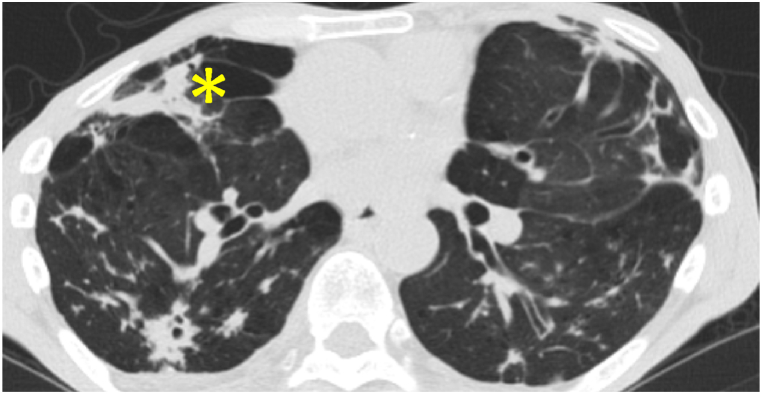
Computed tomography of the chest showing multiple consolidations with cavities, bronchiectasis, and emphysema. *Asterisk* shows a thin-wall cavity of the right middle lobe.

**Fig. 2:**
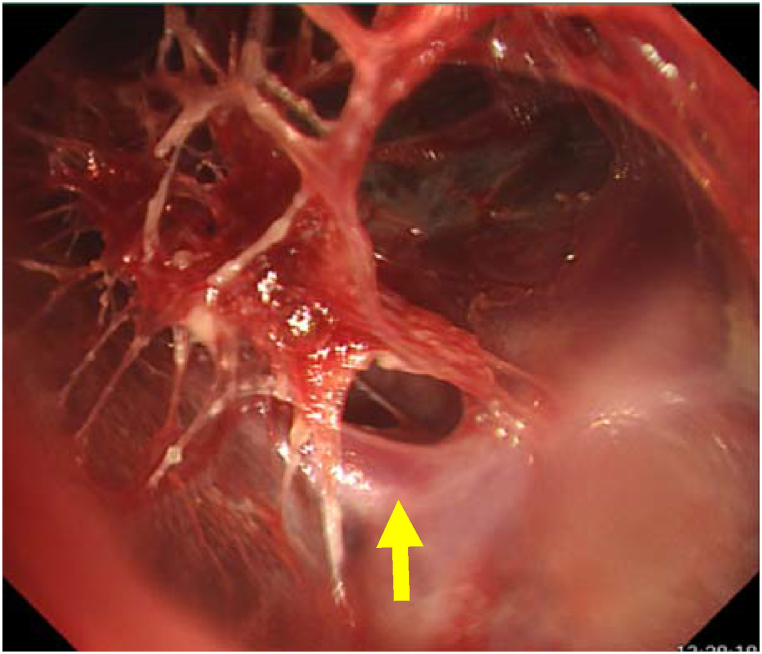
Bronchoscopic view of inside the cavity of the right middle lobe showing a spider-web-appearing architecture consisting of many cord-like tissues loosely adherent to the cavity lumen. *Arrow* shows a small pulmonary vasculature.

A 69-year-old man presented to our hospital with a 1-year history of cough and sputum production. He had a smoking history of 40 pack-years and had been in close contact with six domestic cats. Rhonchi were auscultated throughout both lung fields. Laboratory findings showed elevated white blood cells (11,700/mL, neutrophils 84.1%) and C-reactive protein (9.12 mg/dL). Chest X-ray showed bilateral infiltrative shadows, and a chest computed tomography scan revealed multiple consolidations with cavities, bronchiectasis, and emphysema in both lung fields (Fig. 1). The bacterium isolated in pure culture from his sputum (>10^7^ cfu/mL) was identified biochemically as *Pasteurella multocida* by using an ID test HN-20 Rapid identification kit (Nissui, Tokyo, Japan). Mycobacterium culture was negative. On bronchoscopic evaluation, mild edema with purulent secretions was observed in the segmental and subsegmental bronchi. The flexible bronchoscope (outer diameter 4.9 mm) was navigated through the right middle lobe bronchus (B^5^a), which opened inside the cavity, allowing visualization of a spider-web-appearing architecture consisting of many cord-like tissues loosely adherent to the cavity lumen (Fig. 2). Biopsied tissue specimens from them contained numerous neutrophils, lymphocytes, and thickened alveolar walls with collapsed alveolar space suggestive of a remnant of destroyed lung tissues with inflammation. Using these findings, a diagnosis of cavity formation was made secondary to *Pasteurella multocida* infection complicating bronchiectasis and emphysema. The patient was treated with oral amoxicillin/clavulanate for 42 days with clinical improvement. *Pasteurella multocida* is a commensal of the upper respiratory tract of various animals [1]. Human infection most commonly occurs as soft-tissue infection after animal bites and the second most common respiratory tract infection after inhaling contaminated secretions [2]. *Pasteurella* respiratory infection most commonly occurs in elderly patients with underlying lung diseases, such as pulmonary emphysema and bronchiectasis, as in this patient [2]. Pneumonia and bronchitis are the most common respiratory characteristic; only 3% of patients reportedly had lung abscesses and some of which can be accompanied by cavity formation [[2], [3], [4]]. Bronchoscopic visualization of the inner cavity is extremely unusual because it must be communicated with a dilated bronchus to introduce a bronchoscope. A few bronchoscopic studies showed inside the tuberculous cavity is composed of caseum necrotic debris [5]. In this patient, the underlying bronchiectasis allowed the passage of a bronchoscope into the cavity and visualization of lung architectural destruction. *Pasteurella* infection should be considered as a cause of a lung cavity in patients with chronic lung disease. History taking regarding animal exposure is important for its diagnosis.

## Consent

Written informed consent was obtained from the patient for the publication of this case report and accompanying images. A copy of the written consent is available for review by the Editor-in-Chief of this journal on request.

## Author contribution statement

All authors listed have significantly contributed to the investigation, development and writing of this article.

## Data availability statement

Data will be made available on request.

## Declaration of competing interest

The authors declare that they have no known competing financial interests or personal relationships that could have appeared to influence the work reported in this paper.

## References

[1] Wilson B.A., Ho M. (2013). Pasteurella multocida: from zoonosis to cellular microbiology. Clin. Microbiol. Rev..

[2] Klein N.C., Cunha B.A. (1997). Pasteurella multocida pneumonia. Semin. Respir. Infect..

[3] Seki M., Sakata T., Toyokawa M., Nishi I., Tomono K. (2016). A Chronic respiratory Pasteurella multocida infection is well-controlled by long-term macrolide therapy. Intern. Med..

[4] Lion C., Lozniewski A., Rosner V., Weber M. (1999). Lung abscess due to beta-lactamase-producing Pasteurella multocida. Clin. Infect. Dis..

[5] Mishra D.R., Bhatta N., Acharya A.B., Verma A., Shahi R., Shah N. (2020). Bronchoscopic visualization of a cavity in entirety: an unusual finding. Respirol Case Rep.

